# Editorial: Inclusive health communication: strategies for equitable information dissemination

**DOI:** 10.3389/fpubh.2026.1812907

**Published:** 2026-03-09

**Authors:** Ariadna Munté-Pascual, Laura Ruiz-Eugenio

**Affiliations:** 1 Social Work Training and Research Section, University of Barcelona, Barcelona, Spain; 2 Department of Theory and History of Education, University of Barcelona, Barcelona, Spain

**Keywords:** dialogic approaches, digital health information, inclusive health communication, misinformation, social media dynamics

Health and risk communication is a rapidly evolving field that plays a decisive role in shaping public health outcomes. In recent years, new methodologies have enabled a more precise analysis of how health information circulates and how it is interpreted by different publics and communities. Among these methodological advances, the Social Impact in Social Media (SISM) approach stands out. SISM analysis showed that false health news circulated more widely in Western digital environments than in those analyzed in China; however, science-based evidence was also shared more often in Western contexts than in Chinese ones (1). Additionally, recent applications of SISM have identified distinct interaction patterns depending on the type of content: fake health information elicits more aggressive responses; evidence-based messages with demonstrable social impact generate more respectful and transformative exchanges; and deliberative interactions in social media can help counteract the spread of false information (2).

Although these advances open promising avenues, persistent inequalities affect the capacity of different groups to access, understand, and participate in health communication. Even as more inclusive methodologies emerge, particularly relevant for communities with lower educational and socioeconomic status, there remains a need for strategies that help reduce these gaps and ensure equitable access to evidence-based health information (3).

In this context, this Research Topic examines the multifaceted nature of health and risk communication, with particular attention to how health information is disseminated and received across diverse communities. The contributions are organized around four main thematic axes: (a) the analysis of misinformation, institutional distrust, and the cognitive processes that shape responses to health messages; (b) strategies for adapting health communication to culturally and educationally diverse audiences; (c) the dynamics of health information diffusion on digital platforms and the factors that influence public engagement; and (d) methodological innovations for evaluating, planning, and enhancing health communication interventions.

A first thematic axis addresses misinformation, institutional distrust, and cognitive mechanisms that shape how different publics process health information. The case of Romania's vaccination campaign, presented by Dascalu et al., illustrates how sociopolitical factors, persistent misinformation, and ineffective governmental communication can lead to low vaccine uptake and have serious consequences, such as the subsequent severe measles outbreak. In parallel, Kuang analyses how anti-intellectualism in social media affects scientific communication during health emergencies, showing that digitally amplified distrust toward experts reduces receptivity to evidence-based messages and increases the circulation of misconceptions.

In this line, the study by Gao et al. examines how physicians and patients in China interpret citizen witnessing (non-professional, first-hand reporting) on conflicts in the doctor–patient relationship. Two elements prove decisive for fostering trust or distrust: authority (who speaks and which sources are cited) and witness presence (being physically at the scene and providing direct evidence). When these are absent, uncertainty increases in an already tense clinical environment. Similarly, Le et al. show how social media users handle cognitive conflict when confronted with health information that contradicts prior attitudes. Their results show that low-credibility or low-relevance information triggers information avoidance, while highly relevant and credible information activates information curiosity, leading users to verify content instead of ignoring it.

A second thematic axis focuses on how health communication strategies must adapt to the specific needs of diverse audiences, particularly those with lower educational levels or belonging to minority cultural groups. In this regard, Munté-Pascual et al. present the implementation of Dialogic Scientific Gatherings with non-academic Roma women. Their study shows that creating dialogic spaces in which participants read, discuss, and appropriate scientific evidence about health (selected topics by the women themselves), activates collective meaning-making processes, challenges stereotypes about their supposed disinterest in science and health, strengthens existing health knowledge and practices, and supports more informed decision-making for themselves and their families.

Other studies address groups vulnerable for social or clinical reasons. Mohri et al. analyse how negative attitudes among adults with type 2 diabetes in Japan shape their information-seeking behaviors and disease management. They find that those with poorer glycemic control (higher HbA1c) tend to seek less information, but certain negative patterns (such as self-blame), can, in some cases, stimulate information seeking. For example, individuals who blamed themselves and delayed visiting a physician were later the ones who most actively sought information to better understand their situation. This insight suggests opportunities to adjust communication support for patients who, despite negative emotions, show readiness to engage with information. In parallel, Wang highlights inequalities in oral health among people with disabilities in China and the need for targeted communication and policy interventions. Lee and Lee validate the Korean version of the Sexual Communication Self-Efficacy Scale, confirming its reliability and structure. Their findings demonstrate the scale's utility for assessing self-efficacy in sexual communication (negotiating condom use, expressing boundaries, or discussing sexual history) skills essential for preventing risky sexual behaviors in college student populations.

A third thematic axis examines how health information spreads through digital platforms and which elements facilitate or hinder public engagement. Zhu et al. analyse topic circles in Weibo, identifying how core, intermediate, and peripheral user roles influence the amplification or dissipation of messages during public health incidents. Peripheral users, although numerous, amplify content mainly when it is highly relevant or emotional, whereas core users maintain more stable and structurally influential dissemination patterns. Complementing this line, Yu and Zhao examine over 1,800 posts following the lifting of COVID-19 restrictions in China and show that specific textual features, such as informative language and credibility cues, enhance engagement, while excessive visual elements may have the opposite effect. Xu et al. provide experimental evidence on message framing, demonstrating that loss-social framing (emphasizing the negative consequences for society when people do not comply with preventive measures) is the most effective for increasing compliance intentions. Lastly, Riles et al. investigate how memorable media messages about mental illness, filtered through racial identity cues, influence culpability perceptions and support for public policies. They find that recalling messages featuring perceived racial outgroups elicits more explicit references to race and more negative language, helping explain how such messages may reproduce inequalities.

A fourth thematic axis addresses methodological innovations aimed at improving the evaluation and planning of health communication interventions. Ewers et al. design and preliminarily validate an instrument for assessing disaster health literacy, addressing a pressing need in a field with limited measurement tools. Collantes-Loo and Bravo integrate theoretical models, including the Theory of Planned Behavior, the Protection Motivation Theory, and the Structural Influence Model of health communication, to explain vaccination behavior across population profiles, highlighting moderators such as institutional trust or ease of information processing. Kang et al. present a comprehensive evaluation design for a maternal and child health community intervention in the Philippines, emphasizing the importance of monitoring and implementation quality to strengthen community health systems. Finally, Guo et al. reconceptualize the Balanced Scorecard, traditionally a performance measurement system structured around four perspectives (financial, patient, internal processes, and learning and growth), as a communication tool that articulates values, objectives, and processes in healthcare organizations.

From this diverse set of articles, several cross-cutting contributions emerge that reinforce the idea that inclusive health communication must simultaneously address the cognitive, social, institutional, and technological factors shaping information flows. The studies advance understanding of misinformation and distrust, reveal how social and educational inequalities continue to hinder equitable access to rigorous information, and demonstrate the transformative potential of dialogic, co-creative approaches. They also contribute new methodologies, conceptual models, and tools for assessing individual capacities and the effectiveness of community and organizational interventions.

The implications for public health policy and communication practice are significant. The evidence gathered indicates that communication efforts must be sensitive to cultural, socioeconomic, and cognitive differences, and that the most effective strategies combine scientific rigor, cultural responsiveness, and community participation. The articles underscore the need to strengthen media and health literacy, reinforce institutional trust, and develop policies to mitigate the impact of misinformation in digital environments. They also highlight the value of designing messages tailored to audience motivations and behaviors, fostering dialogue between communities and scientists, and supporting the active role of patients and citizens in knowledge generation.

Looking ahead, further research should examine the long-term effects of inclusive communication strategies, adapt health communication methodologies to increasingly diverse social and cultural contexts, and explore the ethical and equitable use of emerging technologies, including artificial intelligence, to broaden access to reliable health information, particularly among groups experiencing greater inequalities, such as those with low educational attainment.
